# Polyunsaturated and Saturated Oxylipin Plasma Levels Allow Monitoring the Non-Alcoholic Fatty Liver Disease Progression to Severe Stages

**DOI:** 10.3390/antiox12030711

**Published:** 2023-03-13

**Authors:** Miguel D. Ferrer, Clara Reynés, Margalida Monserrat-Mesquida, Magdalena Quetglas-Llabrés, Cristina Bouzas, Silvia García, David Mateos, Miguel Casares, Cristina Gómez, Lucía Ugarriza, Josep A. Tur, Antoni Sureda, Antoni Pons

**Affiliations:** 1 Research Group in Community Nutrition and Oxidative Stress, University of the Balearic Islands-IUNICS, 07122 Palma de Mallorca, Spain; miguel-david.ferrer@uib.es (M.D.F.);; 2 Research Group in Community Nutrition and Oxidative Stress, Health Research Institute of Balearic Islands (IdISBa), 07120 Palma de Mallorca, Spain; 3 CIBER Fisiopatología de la Obesidad y Nutrición (CIBEROBN), Instituto de Salud Carlos III (ISCIII), 28029 Madrid, Spain; 4 Radiodiagnosis Service, Red Asistencial Juaneda, 07011 Palma de Mallorca, Spain; 5 Clinical Analysis Service, University Hospital Son Espases, 07198 Palma de Mallorca, Spain; 6 Camp Redó Primary Health Care Center, 07010 Palma de Mallorca, Spain; 7 Laboratori de Ciències de l’Activitat Física, Universitat de les Illes Balears, 07122 Palma de Mallorca, Spain

**Keywords:** fatty acids, fatty liver, inflammation, metabolic syndrome, oxylipins

## Abstract

Hepatic fat accumulation is the hallmark of non-alcoholic fatty liver disease (NAFLD). Our aim was to determine the plasma levels of oxylipins, free polyunsaturated fatty acids (PUFA) and markers of lipid peroxidation in patients with NAFLD in progressive stages of the pathology. Ninety 40–60-year-old adults diagnosed with metabolic syndrome were distributed in without, mild, moderate or severe NAFLD stages. The free PUFA and oxylipin plasma levels were determined by the UHPLC–MS/MS system. The plasma levels of oxylipins produced by cyclooxygenases, lipoxygenases and cytochrome P450, such as prostaglandin 2α (PGF2α), lipoxinB4 and maresin-1, were higher in severe NAFLD patients, pointing to the coexistence of both inflammation and resolution processes. The plasma levels of the saturated oxylipins 16-hydroxyl-palmitate and 3-hydroxyl-myristate were also higher in the severe NAFLD patients, suggesting a dysregulation of oxidation of fatty acids. The plasma 12-hydroxyl-estearate (12HEST) levels in severe NAFLD were higher than in the other stages, indicating that the hydroxylation of saturated fatty acid produced by reactive oxygen species is more present in this severe stage of NAFLD. The plasma levels of 12HEST and PGF2α are potential candidate biomarkers for diagnosing NAFLD vs. non-NAFLD. In conclusion, the NAFLD progression can be monitored by measuring the plasma levels of free PUFA and oxylipins characterizing the different NAFLD stages or the absence of this disease in metabolic syndrome patients.

## 1. Introduction

Non-alcoholic fatty liver disease (NAFLD) is identified in approximately 10% of children and 20–30% of adults in the Western world [1,2,3,4]. NAFLD is a disease with no well-defined signs or symptoms, with a histological spectrum ranging from fatty liver alone to non-alcoholic steatohepatitis (NASH) [5]. Liver steatosis is the pathologic accumulation of fat inside the hepatocytes (mainly as triglycerides), and it is strongly linked to insulin resistance, obesity and overweight [6]. Patients affected by NAFLD may evolve to NASH, cirrhosis and end-stage liver failure [5]. Hepatic fat accumulation is therefore the hallmark of NAFLD [7]. Grading NAFLD patients provides prognostic information and identifies patients who may benefit from therapy. There are currently no effective pharmacological therapies against NAFLD, insulin resistance being the main pharmacological target [8]. The current interventions include dietary and lifestyle modifications to control body weight, metabolic syndrome and cardio-metabolic risk factors [9]. The reference standard for the diagnosis and grading of hepatic steatosis is liver biopsy [10,11], although magnetic resonance imaging–estimated proton density fat fraction (MI-PDFF) has also been validated to grade the disease [12], and it is preferably used for NAFLD screening in epidemiologic studies [13].

The NAFLD/NASH pathophysiology involves at least two steps. The first step involves insulin resistance and is associated with an increased rate of lipolysis and the release of free fatty acids from adipose tissue, which are available for hepatic uptake and re-esterification to triacylglycerols [14,15,16]. The second step is oxidative stress, which produces lipid peroxidation and activates inflammatory pathways, causing the progression of the pathology to NASH [17]. Lipotoxicity exerts a key role in the pathogenesis of NASH [7,18,19,20,21]. Oxidized low-density lipoproteins (LDL-c), as well as fatty acid oxidized metabolites and other reactive metabolites, are increased in patients with NASH compared with those with NAFLD [16,18,22,23,24,25]. Since most polyunsaturated fatty acids (PUFA) and saturated fatty acids (SFA) oxidized metabolites are endogenous signaling molecules [26], the identification of changes in specific lipid mediators depending on the different degrees of NAFLD might shed light onto the mechanisms contributing to the progression of this disease and reveal novel therapeutic targets and biomarkers.

Oxylipins are a family of peroxidation products of PUFA and SFA with bioactive properties, including inflammation and immune regulatory properties [27]. These compounds are formed via mono- or dioxygen-dependent catalyzed or non-catalyzed reactions [28]. Oxylipin biosynthesis requires cytosolic phospholipase A_2_ (cPLA2)-mediated release of free fatty acids from cell membranes and enzymatic or non-enzymatic oxidation [29,30]. Some of the enzymes that catalyze the oxidation of free PUFA into oxylipins are cyclooxygenases (COXs), lipoxygenases (LOXs) and cytochrome P450 (CYP450) [31,32], which are expressed in a variety of cells and tissues [33]. The action of these enzymes on SFA to synthetize saturated oxylipins is poorly known. CYP450 catalyzes the formation of ω-hydroxide PUFA and SFA in the liver [34]. The β-hydroxy-saturated fatty acids could be synthetized as intermediate products of the β-oxidation of long-chain fatty acids catalyzed by the trifunctional protein (MTP), a multienzyme complex in the inner mitochondrial membrane. The assessment of 3-OH-fatty acids (C14 to C18) in serum is used as a hallmark of MTP deficiency [35]. Hydroxy-saturated fatty acids, such as 12-hydroxystearic acid, are esters of hydroxyl fatty acids (FAHFAs), endogenous lipids that exert anti-inflammatory and anti-diabetic action [36]. The enzymes responsible for FAHFA biosynthesis in vivo remain unknown, although an adipose triglyceride lipase has been identified as being possibly responsible for their biosynthesis [36]. The major pathway for the synthesis of oxylipins are COXs, enzymes which convert PUFAs into isoprostanes, such as prostaglandins (PG) and thromboxanes (Tx). Other oxylipins, such as hydroxy-eicosatetraenoic acids (HETEs) and their derived metabolites, including eoxins, leukotrienes (LTs), lipoxins (LXs), maresins (MaRs), protectins and resolvins (Rv), are synthetized by LOXs.

In recent years, the roles of oxylipins in the inflammatory process have been described [5,15,37]. In this instance, the plasma oxylipin levels are the result of the biosynthesis/degradation balance associated with the inflammation status. Macrophages, neutrophils, mononuclear cells, adipose tissue, muscle and liver can synthetize and secrete oxylipins [38], which are used as autocrine and paracrine signaling molecules for interorgan communication and inflammation management. The hepatic inflammation present in NAFLD is related to macrophage recruitment. In fact, the hepatic recruitment of macrophages promotes the development of NASH [17]. The recruited macrophages are responsible for the production of inflammatory mediators, including oxylipins [39]. In this study, we aimed to measure the plasma levels of polyunsaturated and saturated oxylipins, free PUFA and markers of lipid peroxidation and oxidative stress in patients with NAFLD in progressive stages of severity of the pathology. We also aimed to find the associations of certain plasma free fatty acids and oxylipins and the intrahepatic fat content in NAFLD patients and to estimate the potential diagnostic value of these plasma markers to range NAFLD steatosis.

## 2. Materials and Methods

### 2.1. Design and Participants

Ninety 40–60-year-old adults recruited in the Balearic Islands, Spain, with NAFLD diagnosed by magnetic resonance imaging were selected. The inclusion criteria consisted of meeting at least three of the five metabolic syndrome traits described by the International Diabetes Federation (IDF) consensus [40]: (1) body mass index (BMI) 27–40 Kg/m^2^ or an increased waist circumference of ≥94 cm in men and ≥80 cm in women; (2) triglyceride levels ≥ 150 mg/dL; (3) reduced HDL cholesterol < 40 mg/dL in men and <50 mg/dL in women; (4) increased blood pressure (BP), systolic BP ≥ 130 mmHg or diastolic BP ≥ 85 mmHg; (5) fasting serum glucose level ≥ 100 mg/dL. The following exclusion criteria were applied: liver diseases (other than NAFLD); viral, autoimmune and genetic causes of liver disease; previous cardiovascular disease; active cancer or a history of malignancy in the previous five years; previous bariatric surgery; non-medicated depression or anxiety; pregnancy; primary endocrinological diseases (other than hypothyroidism); alcohol (>21 and >14 units of alcohol a week for men and women, respectively) and drug abuse; weight loss medications in the past six months; concomitant therapy with steroids; inability or unwillingness to give informed consent or communicate with study staff.

All the procedures were approved by the Ethics Committee of the Balearic Islands (ref. IB 2251/14 PI). The study protocol followed the ethical standards of the Declaration of Helsinki. All participants were informed of the purpose and the implications of the study, and all provided written consent to participate. This study was registered in Clinicals Trials.gov ref. NCT04442620 [41].

NAFLD diagnosis was performed by abdominal magnetic resonance imaging–estimated proton density fat fraction (MI-PDFF) (Signa Explorer 1.5T, General Electric Healthcare, Chicago, IL, USA) [42]. Participants were grouped according to their intrahepatic fat content. Four stages of NAFLD were defined according to hepatic steatosis measured as percentage of intrahepatic fat content (IFC): IFC0 (stage 0 or control group without steatosis) IFC < 6.4%; IFC1 (stage 1 with mild steatosis) 6.4% ≤ IFC < 17.4%; IFC2 (stage 2 with moderate steatosis) 17.4% ≤ IFC < 22.1%; and IFC3 (stage 3 with severe steatosis) IFC ≥ 22.1%, following previous criteria for steatosis grade classification [12]. The proportions of hepatocytes containing macrovesicles of fat in these steatosis grades were: grade IFC0 for less than 5%, grade IFC1 for 5–33%, grade IFC2 for 33–66% and grade IFC3 for more than 66% [12].

### 2.2. Anthropometric Characteristics, Blood Collection and Biochemistry Analysis

Weight (kg) was measured with subjects in bare feet and light clothes using calibrated scales. An amount of 0.6 kg for their clothing was subtracted from the total weight. Height (m) was determined with the participant’s head in the Frankfurt plane with a wall-mounted stadiometer (Seca 213, SECA Deutschland, Hamburg, Germany) to the nearest millimeter. Body mass index (BMI) was calculated in kg/m^2^. Blood pressure was measured in triplicate with a validated semi-automatic oscillometer (Omron HEM, 750CP, Hoofddorp, The Netherlands) in a seated position.

Venous blood samples were collected from the antecubital vein in suitable vacutainers with ethylenediaminetetraacetic acid (EDTA) as anticoagulant after 12 h of fasting conditions. Plasma was obtained after centrifugation of the fresh blood at 1700 *g*, 15 min at 4 °C. Biochemical and blood cell parameters, including glucose, glycosylated hemoglobin (Hb1Ac), total cholesterol, high-density lipoprotein cholesterol (HDL-c), LDL-c, triglycerides (TG), alanine aminotransferase (ALT), aspartate aminotransferase (AST), γ-glutamyltransferase (GGT) and platelet count, were determined using standardized clinical procedures [13]. The fibrosis-4 index (FIB-4) was calculated from the data on age, AST and ALT activities and the platelet count according to the following formula:

FIB-4 = (age × AST)/[PLT × (ALT)^1/2^]

where ALT: alanine aminotransferase; AST: aspartate aminotransferase; FIB-4: fibrosis-4 index; PLT: platelet count.

The FIB-4 cut-off values for diagnosing the patients in terms of liver fibrosis were: FIB-4 lower than 1.3, no fibrosis; FIB-4 between 1.3 and 2.67, liver fibrosis; patients with FIB-4 higher than 2.67, severe liver fibrosis [43].

### 2.3. Malondialdehyde Assay

Malondialdehyde (MDA), a plasma marker of peroxidation of PUFAs, was measured using a specific colorimetric assay kit (Sigma-Aldrich Marck^®^, St. Louis, MO, USA), following its instruction manual. The method is based on the reaction of MDA with n-methyl-2-phenylindole, generating a stable chromophore, and measuring the absorbance at 586 nm. Plasma samples and standards were reacted with n-methyl-2-phenylindole in acetonitrile:methanol (3:1) and HCl (12 N) at 45 °C for 1 h. A standard curve of known MDA concentrations was used to calculate the concentration in the plasma samples.

### 2.4. Oxylipin Determination

The plasma oxylipin levels were determined in plasma by an adaptation of a method developed for the simultaneous determination of PUFA and SFA oxylipins in immune cells, based on solid phase extraction (SPE) and HPLC-MS/MS technology, and using deuterated oxylipin (d4-PGF2α) as an internal standard [28].

Oxylipins were purchased from Cayman Chemical (AnnArbor, MI, USA), Santa Cruz Biotechnologies (Finell ST, Dallas, TX, USA) and Sigma Aldrich (Darmstadt, Germany). Cayman Chemical (Ann Arbor, MI, USA) provided (Z)-7-[(1R,2R,3R,5S)-3,5-dihydroxy-2-[(E,3S)-3-hydroxyoct-1-enyl]cyclopentyl]hept-5-enoic acid (PGF2α, Prostaglandin 2a) and deuterated internal standard ProstaglandinF2a-d4 (d4-PGF2α). SigmaAldrich provided: 5,8,11,14-Eicosatetraenoic acid (AA, Arachidonic acid; 16-Hydroxy-hexadecanoic acid (16-hydroxy-palmitic acid, 16−HPAL); 3-Hydroxy-tetradecanoic acid (3-hydroxy-myristic acid, 3-HMYR); 12-Hydroxyoctadecanoic acid (12-hydroxy-stearic acid, 12-HEST). Santa CruxBiotechnology provided: 15-Hydroxy-5,8,11,13-Eicosatetraenoicacid (15-HETE), 17-Hydroxy-4,7,10,13,16,19-docosahexaenoicacid (17-DoHE), Resolvin D2 (RvD2); 5,8,11,14,17-eicosapentenoicacid (EPA); 8,11,14,17-eicosatetraenoic acid (ETA); 7R,14S-dihydroxy-4Z,8E,10E,12Z,16Z,19Z-docosahexaenoic acid (MaR1,Maresin-1); (5S,6E,8Z,10E, 12E,14R,15S)-5,14,15-Trihydroxyicosa-6,8,10,12-tetraenoic acid (LXB4, LipoxinB4); (5S,6Z,8E, 10E,12R,14Z)-5,12-Dihydroxyicosa-6,8,10,14-tetraenoic acid (LTB4, Leukotriene B4).

Strata^®^C-8 cartridge (100 mg, 55 μm, 70 Å from Phenomenex^®^) for solid phase extraction and the analytical column Luna C8 (150 mm × 2.0 mm, 5 μm) were purchased from Phenomenex^®^ (Torrance, CA, USA). Products for HPLC (acetonitrile, methanol, ammonium formiate of HPLC Grade) were purchased from Fisher Scientific (Pittsburgh, PA, USA).

Oxylipins and free PUFAs were extracted from plasma after the addition of deuterated d4-PGF2α at a final concentration of 1 ng/mL as internal standard, following a procedure previously described [28]. The oxylipins and free fatty acids were extracted and concentrated with a Strata^®^C-8 cartridge (previously washed with 1 mL of formic acid 0.1%) connected to a Visiprep SPE vacuum manifold (Supelco Co., St. Louis, MO, USA). Samples with internal standards were prepared by diluting 1:2 with formic acid 0.1% (0.5 mL of the sample and 1 mL of formic acid) and centrifuged at 1200× *g* to eliminate the proteins. The deproteinized samples where then eluted trough the column. After elution, the columns were washed with 1 mL of 0.1% formic acid, and the analytes were finally eluted with 1 mL of methanol. The collected eluate was evaporated in an Eppendorf Concentrator 5301^®^ at 30 °C; the residues were dissolved in 50 μL of 50% methanol and injected into the LC–MS/MS system.

A UHPLC system (Ultimate 3000, Thermo Fisher Scientific, Waltham, MA, USA) coupled to a Q-Exactive Hybrid Quadrupole-Orbitrap mass spectrometer (ThermoFisher^®^Scientific, Waltham, MA, USA) operating with a heated electrospray interface (HESI) was employed. Spectra were recorded in negative mode. The analytical column was Luna C8 (150 mm × 2.0 mm, 5 μm; Phenomenex, Torrance, CA, USA) maintained at 40 °C. The mobile phases used in this separation were 0.5 mM ammonium formiate (pH 3.3) (A) and acetonitrile with 0.5 mM ammonium formiate (B). The stepwise linear gradient was 5–35% B from 0 min to 5 min, 35–65% B from 5 to 15 min, 65–75% from 15 to 20 min, 75–100% B from 20 to 24 min and held at 100% B from 24 to 28 min. The system was returned to initial conditions from 100 to 5% B from 28 to 29 min and held at 5% until 34 min to equilibrate the column. A flow rate of 0.3 mL/min and injection volume of 10 μL were used. The temperature of ion transfer capillary, spray voltage, sheath gas flow rate, auxiliary gas flow rate and S-lens RF level were set to 350 °C, 3.1 kV in negative mode, 35 arbitrary units (AU), 10 AU and 55 AU, respectively. Full scan acquisition was performed over a range of 150–700 m/z with a resolution of 70,000. During the MS/MS scans, precursors were fragmented with a normalized collisional energy of 60 AU. Ions were selected for MS/MS analysis from an inclusion list in accordance with the m/z found by using each standard oxylipin and free fatty acid. XcaliburTM4.1, Trace Finder 4.1 SP2 software were used for data processing (Thermo Fisher Scientific).

The concentrations of fatty acids and oxylipins in plasma samples were calculated using deuterated d4-PGF2α as internal standard. Pure commercial free fatty acids or oxylipins were used to analyze the individual response of each oxylipin and free fatty acid with respect to the d4-PGF2α internal standard. A mixture of these oxylipins and fatty acids and deuterated d4-PGF2α internal standard at 50 ng/mL in water was processed together with the plasma samples. The differential response of each oxylipin or fatty acid with respect to the d4-PGF2α internal standard (internal standard factor, ISF) was calculated with the LC–MS/MS signal. The ISFs were calculated following the formula previously described [28]. Once the ISF had been calculated, this value was used to calculate the oxylipin or fatty acid concentration in the plasma following the procedure previously described [28].

### 2.5. Statistical Analysis

Statistical analysis was carried out using the Statistical Package for Social Sciences (SPSS v.21.0 for Windows). The results were expressed as mean ± SEM, and *p* ≤ 0.05 was considered statistically significant. A Kolmogorov–Smirnov test was applied to assess the normal distribution of the data. Data were normally distributed, and the statistical significance was assessed by a one-way analysis of variance (ANOVA) using the steatosis degree as a statistical factor. When a significant effect of the ANOVA was found, a Bonferroni post hoc test was performed to identify differences between the groups. The correlations between plasma oxylipin and fatty acid concentrations and between IFC and MDA, oxylipin and fatty acid concentrations were calculated using the bivariate correlation Pearson test. The discriminatory capability of plasma oxylipin or fatty acid concentrations for different steatosis grades was tested by using the following dichotomizations: IFC0 vs. IFC1 or greater; IFC1 or less vs. IFC2 or greater; IFC2 or less vs. IFC3. For each set of dichotomized steatosis grades, the area under the receiver operating characteristic curve (AUCROC) was calculated. The oxylipins or fatty acids with an AUCROC significantly different from 0.5 were selected as parameters with discriminant capability. The lowest plasma oxylipin or fatty acid threshold value that provided a specificity to distinguish between dichotomized steatosis grades equal or greater than 90% was selected. At that oxylipin or fatty acid threshold value, the raw sensitivity, specificity, accuracy, positive predictive value and negative predictive value to distinguish between dichotomized steatosis grades were calculated.

## 3. Results

A total of 90 metabolic syndrome patients underwent MI-PDFF and were classified into the different hepatic steatosis degrees. The IFC of 19 patients was lower than 6.4% (IFC0, without NAFLD); 42 patients presented an IFC between 6.4% and 17.4% (IFC1, NAFLD grade 1); 19 patients presented an IFC value between 17.4% and 22.1% (IFC2, NAFLD grade 2); and 10 patients presented an IFC value higher than 22.1% (IFC3, NAFLD grade 3). No differences in BMI were observed between IFC0, IFC1, IFC2 and IFC3 groups, all these values being indicative of obesity (Table 1). The mean values of systolic and diastolic blood pressure and total and LDL-c in serum were indicative of hypertension and hypercholesterolemia in all groups, with no differences observed between groups. The circulating triglycerides, glucose and HbA1 in the IFC3 group were significantly higher than in the other groups. The HDL-c levels were significantly higher in the IFC0 group compared to the other groups. The plasma MDA levels, indicative of degradation of PUFA by peroxidation, were increased in the IFC2 group compared to the IFC0 group. The markers of hepatic function were affected differently. AST was not affected by the degree of steatosis. However, patients with IFC2 and IFC3 presented increased ALT activity compared to patients with IFC0 and IFC1. Patients with IFC3 presented higher GGT activity than patients with IFC0. GGT activity in patients with IFC1 and IFC2 was no different to that of patients with IFC0 and IFC3. The number of patients without liver fibrosis (FIB-4 < 1.3) was 71, while 19 patients presented liver fibrosis (FIB-4 ≥ 1.3). Of the patients with liver fibrosis, only four had severe fibrosis (FIB-4 ≥ 2.67). The 19 patients who presented liver fibrosis were distributed between the different IFC groups without a clear pattern (5 patients in the IFC0 group, 7 patients in the IFC1 group, 5 patients in the IFC2 group and 2 patients in the IFC3 group). The FIB-4 mean values in all steatosis groups were lower than 1.3, and there were no statistical differences between groups. An effect of the degree of liver steatosis was observed in the plasma levels of MDA. The three groups with steatosis (IFC1, IFC2 and IFC3) presented equivalent MDA levels, but only in the IFC2 group were these levels significantly increased compared to the IFC0 group.

The NAFLD grade significantly influenced the plasma levels of AA, EPA, ETA, MaR1, LXB4, 3HMYR, 16HPAL, 12HEST and PGF2α (Table 2), whereas the 15HETE, 17DoHE, RvD2 and LTB4 plasma levels were not affected by the degree of steatosis. The plasma free fatty acids and oxylipin levels of IFC0 were similar to those of IFC1 and IFC2, which were also similar between them. The main feature observed was the significantly higher AA, EPA, ETA, Mar1, LXB4, 3HMYR, 16HPAL, 12HEST and PGF2α plasma levels in IFC3 NAFLD patients compared to the other groups. The IFC was significantly correlated with 12HEST, 17HDoHE, 15HETE, AA, EPA, 16HPAL, MaR1, ETA, 3HMYR, LXB4, PGF2α and MDA plasma levels (Table 3). The MDA plasma levels were significantly correlated with MaR1, 15HETE, 12HEST, 16HPAL, 3HMYR, RvD2, LTB4 and AA plasma levels.

The correlations between the circulating levels of all the free fatty acids and oxylipins are shown in Table 4. All correlations between plasma free fatty acids and oxylipins were positive; high levels of the fatty acids were associated with high levels of their corresponding oxylipin metabolite. Additionally, the free AA, EPA and ETA plasma levels were significantly correlated between them (Table 4). In summary, free AA plasma levels correlated with 16HPAL, 12HEST, 15HETE, MaR1, 17HDoHE and 3HMYR. Free EPA plasma levels correlated with 16HPAL, 12HEST, MaR1 and 3HMYR. 15HETE plasma levels were correlated with 17HDoHE, 12HEST, MaR1, LXB4, 3HMYR, PGF2α, 16HPAL and AA. The 17DoHE plasma levels were correlated with 15HETE, MaR1, 12HEST, LTB4, LXB4, 3HMYR, PGF2α and 16HPAL. RvD2 plasma levels correlated with 3HMYR, 16HPAL, PGF2α ¡, MaR1, LXB4 and 12HEST. The MaR1 plasma levels were correlated with LXB4, 3HMYR, 16HPAL, 12HEST, PGF2α, 15HETE, 17DoHE, AA, EPA, RvD2 and LTB4. LXB4 plasma levels were correlated with PGF2α, 3HMYR, 12HEST, 16HPAL, RvD2, 15HETE, 17HDoHE and LTB4. The LTB4 plasma levels were correlated with 17HDoHE, 12HEST, MaR1, PGF2α, 3HMYR, LXB4 and 16HPAL. The 3HMYR plasma levels were correlated with PGF2α, LXB4, 12HEST, 16HPAL, Mar1, RvD2, free AA and EPA, 15HETE, 17HDoHE and LTB4. The plasma levels of 16HPAL were correlated with free AA and EPA, Mar1, LXB4, 3HMYR, PGF2α, free ETA, RvD2, 12HEST, 15HETE, 17HDoHE and LTB4. The 12HEST plasma levels were correlated with 3HMYR, LXB4, PGF2α, MaR1, 15HETE, 17HDoHE, LTB4 and 16HPAL. PGF2α plasma levels were correlated with 3HMYR, LXB4, Mar1, 16HPAL, 12HEST, RvD2, LTB4, 15HETE and 17HDoHE.

The raw estimates of accuracy, true positive predictive value and false positive predictive value to detect specific grades of steatosis through plasma free fatty acids or oxylipin levels were calculated by using receiver operating characteristic curve analysis (ROC) (Table 5). The plasma levels of 12HEST, PGF2α, 15HETE and free ETA showed certain diagnostic accuracy in distinguishing patients with different grades of steatosis. The plasma levels of 12HEST allowed differentiating patients with IFC0 or IFC1 grades from patients with IFC2 or IFC3 grades with an AUROC of 0.661 (95% confidence interval: 0.537, 0.788). A plasma concentration of 12HEST greater than 12.3 nM allowed diagnosing IFC2 or IFC3 hepatic steatosis with 83% of true positives, although 72% of false positives were also diagnosed in this category (patients with steatosis grade IFC0 or IFC1 who were diagnosed with grade IFC2 or IFC3). Similarly, 12HEST plasma concentration allowed differentiating patients with IFC0 or IFC1 or IFC2 grades from patients with IFC3 grade with an AUROC of 0.694 (95% confidence interval: 0.477, 0.910). A plasma concentration of 12HEST greater than 30 nM allowed diagnosing IFC3 degree hepatic steatosis with 80% of true positives (IFC3 patients who were correctly diagnosed with IFC3 degree), although 53% of false positives were also diagnosed in this category (patients with steatosis grade IFC0, IFC1 or IFC2 who were diagnosed with grade IFC3). The plasma levels of PGF2α had similar sensitivity but more specificity than the 12HEST plasma levels in diagnosing IFC3 steatosis. The PGF2α plasma levels allowed diagnosing IFC3 patients with respect to IFC0, IFC1 or IFC2 patients with an AUROC value of 0.748 (95% confidence interval: 0.537, 0.958). A plasma concentration of PGF2α greater than 0.675 nM allowed diagnosing IFC3 hepatic steatosis with 80% of true positives, with only 17% of false positives. The plasma levels of 15HETE presented a diagnostic value similar to PGF2α in distinguishing IFC3 patients from IFC0, IFC1 or IFC2 patients. Finally, the plasma levels of free ETA also had a diagnostic value in distinguishing patients without hepatic steatosis from those with hepatic steatosis (AUROC value of 0.647; 95% confidence interval: 0.525–0.768). Free ETA plasma levels greater than 0.42 nM indicated hepatic steatosis IFC1, IFC2 or IFC3 degree, with 82% of true positives but 79% of false positives detected.

## 4. Discussion

NAFLD, defined as steatosis affecting ≥ 5% of hepatocytes, progresses by increasing the hepatic fat content through different severity stages until advanced forms of liver injury are reached. The clinical progression of NAFLD is associated with fibrosis, cirrhosis and hepatocellular carcinoma. A pathohistological grading system for hepatic steatosis depending on the percentage of hepatocytes with fatty infiltration has been developed [44,45]. Hepatic steatosis, in the absence of inflammation and fibrosis, is visualized as a general benign state, which is in accordance with the similar values of glycemia, triglyceridemia, cholesterol levels and blood pressure observed in this study between the IFC0, IFC1 and IFC2 stages. However, the IFC3 severe steatosis stage is associated with higher values of glycaemia and triglyceridemia, which points to the pathological relevance of the high fat accumulation in the hepatocytes. The higher glycemia observed in the group of patients with severe NAFLD probably evidences a worsening of insulin resistance at this stage of the disease. The metabolic pathways involved in NAFLD progression are not well characterized, but low-grade chronic inflammation and oxidative stress take part in the pathogenesis and progression of NAFLD [46,47]. MDA, the PUFA peroxidation marker of oxidative stress [48], is progressively increased with the stage of NAFLD, and its plasma levels are significantly correlated with the IFC, reflecting that low-grade oxidative stress is secondary to the accumulation of fatty acids in the liver. The activities of hepatic markers, such as AST, ALT and GGT, were in accordance with previously reported data, which indicate that ALT is commonly increased in NAFLD patients [49], and the AST/ALT ratio is below 0.8 in these patients [50]. GGT is another biochemistry marker, which is usually increased in NAFLD patients, and the results obtained in this study confirm that patients categorized in groups with higher levels of steatosis also present higher GGT activity and are therefore at a more advanced stage of the disease. Regarding the degree of fibrosis, the subjects with NAFLD included in this study did not present fibrosis or presented incipient fibrosis, as pointed out by the low values of the FIB-4 index. These results confirm that the FIB-4 index presents high accuracy and negative predictive value for ruling out advanced liver fibrosis but is more inaccurate in discriminating incipient liver fibrosis stages [51].

The plasma oxylipin profile is associated with oxidative stress and inflammation, since certain oxylipins are the result of enzymatic or non-enzymatic lipid peroxidation, and oxylipins have pro- or anti-inflammatory capabilities [7,27]. The main finding of this study is that the plasma oxylipin levels are influenced by the degree of steatosis in NAFLD patients. The plasma levels of AA, EPA, ETA, MaR1, LXB4, 3HMYR, 16HPAL, 12HEST and PGF2α were higher in patients with severe NAFLD (IFC3) than in patients without or with mild or moderate NAFLD. Severe NAFLD is associated with higher insulin resistance than the previous stages, which can result in increased lipolysis and release of free fatty acids [10,40,42] into circulation, as we observed for AA, EPA and ETA. Oxylipins derived from AA through the cyclooxygenase pathway (COX-1 and COX-2), such as PGF2α, or through the lipoxygenase pathway (5-LOX, 12-LOX and 15-LOX), such as LXB4, were higher in the plasma of severe NAFLD patients compared to the other groups, although 15HETE and LTB4 plasma levels, also derived from AA through lipoxygenases, were similar in all NAFLD stages. These results could indicate the enhanced activity of COX and LOX enzymes in the severe stage of NAFLD. Similar trends for most oxylipins derived from AA have been described comparing NAFLD subjects to healthy controls [52] and in NASH patients compared to NAFLD patients [7,28], evidencing the associations between the activation of LOX pathways and the progression of the disease to NASH. On the contrary, oxylipins derived from docosahexaenoic acid (DHA) through the LOX pathways, such as 17DoHE and RvD2, are present in the plasma of all patients at a similar concentration, although MaR1 plasma levels, also derived from DHA, are increased in severe NAFLD patients compared to the other NAFLD stages. Severe NAFLD is therefore related to the increased production of oxylipins from AA and DHA, such as PGF2α, LXB4 or MaR1, which can exert pro- and anti- or resolving inflammatory activities, respectively. PGF2α increases vascular permeability, recruits neutrophils to sites of injury and promotes the switch from leukotriene biosynthesis to specialized pro-resolving mediators (SPM) production in neutrophils [53,54]. MaR1 and LXB4 are SPM, which counter-regulate dysregulated resolution responses and moderate the proinflammatory phenotype of inflammatory diseases [55,56,57]. The levels of PGF2α, LXB4 and MaR1 are increased in the plasma of severe NAFLD patients, evidencing the activation of both pro-inflammatory and pro-resolving processes at this stage of liver steatosis.

Severe NAFLD patients also have increased plasma levels of saturated oxylipins, such as 3HMYR, 16HPAL and 12HEST. 16HPAL is synthetized from palmitic acid by CYP450 [58,59]. The ω-oxidation of monocarboxylic fatty acids generates the corresponding ω-hydroxycarboxylic acid and takes place predominantly in the kidney and liver. The higher 16HPAL levels observed in IFC3 compared to the other NAFLD stages could indicate an activation of the CYP450 pathway related to inflammation in this severe stage of NAFLD [60]. The increased 16HPAL levels could also be indicative of a decreased function of the peroxisomal ω-oxidation of fatty acids in the severe NAFLD stage. In fact, impaired peroxisomal PUFA metabolism is associated with the progression to NASH [7]. Under normal physiological conditions, the peroxisomal ω-oxidation pathway accounts for 5–10% of total fatty acid oxidation [58]. Peroxisomal ω-oxidation of fatty acids may function as an escape route to overwhelmed mitochondrial β-oxidation [61]. Impairment of β-oxidation pathway leads to the accumulation of β-hydroxy-fatty acids and the reduction in energy production [62]. In this sense, the increased levels of the β-hydroxy-fatty acid 3HMYR in the IFC3 NAFLD stage could indicate a dysfunction of the β-oxidation pathway. The 12HEST biosynthesis pathway has not been described yet [63], although the most probable source for this molecule might be its non-enzymatic acyl chain oxidation by different active forms of oxygen [64]. 12HEST is a component of branched fatty acid esters of hydroxy-fatty acids (FAHFAs), which are endogenous lipids with anti-inflammatory and anti-diabetic action [36]. FAHFA biosynthesis in vivo could be attributed to an adipose triglyceride lipase catalyzing the transacylation reaction, which esterifies hydroxyl fatty acids with a fatty acid from a triglyceride or a diglyceride to produce FAHFAs [36]. The increased 12HEST levels in severe NAFLD patients could be related to increased production of ROS, inducing oxidative stress and enzymatic and non-enzymatic lipid peroxidation. This could also indicate decreased 12HEST consumption to synthetize FAHFAs and, consequently, a low rate of FAHFAs synthesis at this stage of severe NAFLD. Some FAHFAs improve both insulin sensitivity and glucose tolerance in mice by enhancing insulin secretion, glucose transport and insulin action and reducing adipose tissue inflammation [37]. Reducing the rate of FAHFAs production, as indicated by the high levels of 12HEST, could therefore contribute to the progression of NAFLD.

Intrahepatic lipid saturation predisposes the liver to inflammation; however, the mechanisms by which this intrahepatic lipid saturation promotes NAFLD progression are still poorly understood [46]. The obtained data show that the plasma levels of 12HEST and 17HDoHE are the lipid mediators with the highest correlation with the intrahepatic fat content in NAFLD patients. The biosynthesis of these lipid mediators might be enhanced in a situation of intrahepatic lipid saturation. Both liver-resident cells (e.g., Kupffer cells, hepatic stellate cells, sinusoidal endothelial cells) and recruited immune cells (e.g., monocytes, macrophages, dendritic cells, natural killer cells) have metabolic pathways to synthetize pro-, anti- or resolving inflammatory signals [65]. Intrahepatic lipid saturation is related to the activation of 17HDoHE biosynthesis by liver 15-LOX. The 12HEST biosynthesis pathway might involve the non-enzymatic oxidation of stearate by the hydroxyl radical, directly generating the hydroxyl fatty acid 12HEST, since other ROS do not have the ability to react directly with SFA. Non-enzymatic fatty acid oxidation is associated with NAFLD progression to NASH [7]. In this study, we confirmed that the intrahepatic lipid saturation in NAFLD patients is associated with oxidative damage in lipids and with hydroxyl fatty acid production. 

The free fatty acid and oxylipin metabolism is reflected in the correlations found between the plasma levels of free fatty acid and oxylipins. AA, ETA and EPA plasma levels are positively correlated between them, evidencing a common origin and destiny. The plasma levels of 15HETE and 17HDoHE are well correlated, pointing to the role of the same 15-LOX responsible for their synthesis from AA or DHA, respectively. The plasma levels of PGF2α, LXB4 and MaR1 are also positively well correlated, suggesting a common origin, although PGF2α exhibits pro-inflammatory, whereas LXB4 and MaR1 exhibit anti-inflammatory or resolving activities [66]. The synthesis of SPM (such as MaR1 and LXB4) by neutrophils is a pivotal process for the transition from inflammation to resolution [67,68]. SPM, in turn, counter-regulate the proinflammatory mediators, such as PGF2α [66]. The plasma levels of 16HPAL, 3HMYR and 12HEST are well correlated between them and with the plasma levels of PGF2α, LXB4 and MaR1. The origin of these oxylipins is different, as they are synthetized by the action of COX, LOX, CYP450, peroxisomal ω-oxidation, mitochondrial β-oxidation or non-enzymatic reactions with the hydroxyl radical. The high degree of correlation between these oxylipins could be related to their degradation pathway. Oxylipins are biological mediators that require strict control, and only recently, it was started being described how they are removed during inflammation [33]. It seems that oxylipins are removed via mitochondrial β-oxidation. Many oxylipins are removed by carnitine palmitoyl transferase, a mitochondrial importer of fatty acids driving toward β-oxidation [33]. The positive correlation of the plasma concentration of these oxylipins in NAFLD could be attributed to the difficulties in removing them by an overwhelmed β-oxidation pathway.

There is a lack of conclusive biomarkers for the non-invasive monitoring of NAFLD in humans [69]. NASH progression is based upon the NAFLD Activity Score (NAS) and fibrosis using liver biopsies. The Pathology Committee of the NASH Clinical Research Network designed NAS as a scoring system of 14 histological features [44]. Patients are diagnosed as ‘‘NASH’’ with a NAS ≥ 5, while they are diagnosed as “Not NASH’’ with a NAS ≤3. The MI-PDFF represents the standard gold reference for grading “Not NASH” NAFLD in four stages, based on the IFC [12]: without NAFLD, mild NAFLD, moderate NAFLD and severe NAFLD. The plasma content of 11,12-dihydroxyeicosatrienoic acid (11,12-diHETrE) was proposed as a single biomarker to differentiate NAFLD from NASH, with an AUCROC area of 1. A panel of other oxylipins, including 13,14-dihydro-15-keto prostaglandin D2 (dhk PGD2) and 20-carboxy arachidonic acid (20-COOH AA), was also proposed for the diagnosis of NASH [34]. However, no oxylipins have been proposed to date in order to differentiate the stage of “Not NASH” NAFLD. Here, we propose that the free fatty acids and oxylipin plasma levels correlating with the IFC could have diagnostic value to range NAFLD steatosis. We tested the discriminatory capability of circulating oxylipins as biomarkers to differentiate patients between different NAFLD grades. The plasma levels of 12HEST, PGF2α, 15HETE and free ETA showed significant diagnostic accuracy in distinguishing patients with different NAFLD grades. Among all the oxylipins analyzed, 12HEST is the most sensible biomarker to diagnose severe NAFLD (IFC3) but with low specificity. The PGF2α plasma levels exhibit similar sensitivity but higher specificity than 12HEST. The 15HETE plasma levels have a similar diagnostic value as PGF2α in diagnosing severe NAFLD (IFC3), and the ETA plasma levels could be useful in diagnosing a certain degree of NAFLD (IFC1, IFC2 or IFC3) against the absence of NAFLD (IFC0).

In summary, NAFLD progression can be monitored by measuring the plasma levels of free PUFA and oxylipins in metabolic syndrome patients. Severe NAFLD stage is characterized by higher glycemia, triglyceridemia and plasma levels of free PUFA compared to absent, mild or moderate NAFLD, evidencing increased insulin resistance. The plasma levels of oxylipins produced by COX, LOX and CYP450 enzymes (PGF2α, LXB4, MaR1) are higher in severe NAFLD patients than in patients with mild and moderate NAFLD or in patients without NAFLD, pointing to the coexistence of both inflammation and resolution processes associated with this severe stage of the disease. The plasma levels of saturated oxylipins 16HPAL and 3HMYR are higher in severe NAFLD than in the preliminary stages of the disease, which could be indicative of dysregulation of both the mitochondrial β-oxidation and the peroxisomal ω-oxidation pathways. Plasma 12HEST levels in severe NAFLD are higher than in the other stages, indicating that the non-enzymatic hydroxylation of saturated fatty acid produced by activated oxygen species is more present in this severe stage of NAFLD. Finally, the plasma levels of 12HEST and PGF2α could be considered as novel potential biomarkers for diagnosing the severe, moderate or mild stages of NAFLD.

## 5. Conclusions

NAFLD progression can be monitored by measuring the plasma levels of free PUFA and oxylipins in metabolic syndrome patients. The severe NAFLD stage is characterized by increased insulin resistance, dysregulation of both the mitochondrial β-oxidation and the peroxisomal ω-oxidation pathways and the coexistence of both inflammation and resolution processes associated with the severe stage of the disease.

## Figures and Tables

**Table 1:** Anthropometric, diagnostic and biochemistry parameters of the non-alcoholic fatty liver disease patients.

	IFC0 (n = 19)	IFC1 (n = 42)	IFC2 (n = 19)	IFC3 (n = 10)	ANOVA *p*
Age (years)	54.8 ± 1.4 ^ab^	54.3 ± 1.1 ^a^	48.6 ± 1.2 ^b^	52.2 ± 1.5 ^ab^	0.030
Body Weight (Kg)	88.7 ± 2.0 ^a^	92.1 ± 1.7 ^a^	93.8 ± 1.7 ^a^	95.8 ± 3.0 ^a^	0.196
BMI (Kg/m^2^)	32.3 ± 0.5 ^a^	32.6 ± 0.5 ^a^	34.2 ± 0.6 ^a^	33.2 ± 0.8 ^a^	0.132
IFC(%)	4.8 ± 0.2 ^a^	10.0 ± 0.4 ^b^	18.6 ± 1.0 ^c^	32.7 ± 2.1 ^d^	<0.001
Systolic Blood Pressure (mmHg)	132 ± 2.1 ^a^	135 ± 1.8 ^a^	138 ± 2.8 ^a^	143 ± 3.7 ^a^	0.107
Diastolic Blood Pressure (mmHg)	79.5 ± 2.1 ^a^	81.0 ± 1.0 ^a^	81.6 ± 1.6 ^a^	86.7 ± 2.5 ^a^	0.078
Glucose (mg/dL)	102 ± 3.6 ^a^	109 ± 4.0 ^a^	106 ± 2.8 ^a^	141 ± 13 ^b^	<0.001
HbA1 c (%)	5.7 ± 0.1 ^a^	5.9 ± 0.1 ^a^	5.8 ± 0.1 ^a^	6.6 ± 0.3 ^b^	0.002
Triglycerides (mg/dL)	130 ± 8.6 ^a^	199 ± 18 ^b^	191 ± 22 ^ab^	218 ± 32 ^ab^	0.010
HDL cholesterol (mg/dL)	48.7 ± 1.6 ^a^	43.1 ± 1.3 ^b^	42.6 ± 1.5 ^ab^	40.2 ± 1.9 ^b^	0.004
LDL cholesterol (mg/dL)	133 ± 4 ^a^	126 ± 4 ^a^	135 ± 5 ^a^	115± 9 ^a^	0.161
Cholesterol (mg/dL)	207 ± 6 ^a^	207 ± 5 ^a^	214 ± 6 ^a^	219 ± 21 ^a^	0.701
AST (U/L)	25.4 ± 4.1 ^a^	24.8 ± 1.1 ^a^	26.4 ± 3.2 ^a^	28.4 ± 2.0 ^a^	0.790
ALT (U/L)	27.1 ± 4.7 ^a^	28.3 ± 1.9 ^a^	42.0 ± 7.3 ^ab^	49.0 ± 7.7 ^b^	0.002
AST/ALT ratio	1.06 ± 0.09 ^a^	0.99 ± 0.06 ^a^	0.78 ± 0.11 ^a^	0.75 ± 0.14 ^a^	0.097
GGT (U/L)	26.5 ± 3.8 ^a^	35.8 ± 3.3 ^ab^	39.5 ± 5.6 ^ab^	51.0 ± 5.8 ^b^	0.010
Platelets (counts/nL)	232 ± 8 ^a^	232 ± 7 ^a^	268 ± 19 ^a^	250 ± 58 ^a^	0.102
FIB-4	1.24 ± 0.14 ^a^	1.14 ± 0.07 ^a^	0.78 ± 0.09 ^a^	0.98 ± 0.12 ^a^	0.056
MDA (nM)	1.21 ± 0.15 ^a^	1.67 ± 0.14 ^ab^	1.98 ± 0.21 ^b^	2.02 ± 0.38 ^ab^	0.038

Results represent mean ± SEM. Statistical analysis: One-way ANOVA for normally distributed data. When a significant effect of the ANOVA was found, a Bonferroni test was performed to identify differences between groups. Different letters (a, b, c, d) indicate significant differences between groups (*p* < 0.05). ALT: alanine aminotransferase; AST: aspartate aminotransferase; BMI: body mass index; FIB-4: liver fibrosis-4 index; GGT: γ-Glutamyltransferase; HbA1c: glycosylated hemoglobin; HDL: high-density lipoprotein; IFC: intrahepatic fat content; LDL: low-density lipoprotein; MDA: malondialdehyde. IFC0 (stage 0 or control group without steatosis) IFC < 6.4%; IFC1 (stage 1 with mild steatosis) 6.4% ≤ IFC < 17.4%; IFC2 (stage 2 with moderate steatosis) 17.4% ≤ IFC < 22.1%; and IFC3 (stage 3 with severe steatosis) IFC ≥ 22.1%.

**Table 2:** Oxylipin and free fatty acid plasma levels of patients with or without non-alcoholic fatty liver disease (NAFLD) according to the intrahepatic fat content (IFC).

	IFC0 (n = 19)	IFC1 (n = 42)	IFC2 (n = 19)	IFC3 (n = 10)	ANOVA *p*
AA (nM)	67.7 ± 11.9 ^a^	105 ± 15 ^a^	187 ± 57.7 ^a^	765 ± 455 ^b^	0.002
EPA (nM)	14.4 ± 4.86 ^a^	15.2 ± 4.3 ^a^	39.8 ± 23.8 ^a^	194 ± 131 ^b^	0.006
ETA (nM)	0.98 ± 0.18 ^a^	1.76 ± 0.26 ^a^	1.91 ± 0.43 ^a^	13.5 ± 9.6 ^b^	0.008
17DoHE (nM)	2.39 ± 1.14 ^a^	1.00 ± 0.20 ^a^	1.58 ± 0.73 ^a^	3.72± 1.89 ^a^	0.122
RvD2 (nM)	3.19 ± 2.05 ^a^	3.02 ± 0.95 ^a^	1.18 ± 0.48 ^a^	3.68 ± 2.45 ^a^	0.689
MaR1 (nM)	0.21 ± 0.08 ^ab^	0.11 ± 0.02 ^a^	0.17 ± 0.06 ^ab^	0.63 ± 0.41 ^b^	0.030
15HETE (nM)	0.57 ± 0.19 ^a^	0.56 ± 0.09 ^a^	1.01 ± 0.45 ^a^	1.53 ± 0.56 ^a^	0.089
LXB4 (nM)	18.8 ± 11.8 ^a^	9.24 ± 3.32 ^a^	24.1 ± 16.6 ^ab^	203 ± 163 ^b^	0.018
LTB4 (nM)	0.57 ± 0.17 ^a^	1.68 ± 0.61 ^a^	2.60 ± 1.28 ^a^	3.46 ± 1.67 ^a^	0.263
PGF2α (nM)	0.96 ± 0.51 ^a^	0.80 ± 0.45 ^a^	2.32 ± 1.2 ^ab^	12.1 ± 9.7 ^b^	0.023
3HMYR (nM)	90.0 ± 47.9 ^ab^	113 ± 55.7 ^a^	183 ± 98.0 ^ab^	955 ± 746 ^b^	0.035
16HPAL (nM)	69.0 ± 27.5 ^a^	63.5 ± 26.6 ^a^	77.4 ± 53.3 ^a^	785 ± 461 ^b^	0.001
12HEST (nM)	59.3 ± 21.1 ^a^	41.0 ± 6.3 ^a^	140 ± 68.0 ^ab^	345 ± 163 ^b^	0.002

Results represent mean ± SEM. Statistical analysis: One-way ANOVA for normally distributed data. When a significant effect of the ANOVA was found, a Bonferroni test was performed to identify differences between groups. Different letters (a, b) indicate significant differences between groups (*p* < 0.05). AA: 5,8,11,14-Eicosatetraenoic acid (Arachidonic acid); EPA: 5,8,11,14,17-eicosapentenoic acid; ETA: 8,11,14,17-eicosatetraenoic acid; 15HETE: 15-Hydroxy-5,8,11,13-Eicosatetraenoic acid; 17DoHE: 17-Hydroxy-4,7,10,13,16,19-docosahexaenoic acid; RvD2: 7S,16R,17S-trihydroxy-4Z,8E,10Z,12E,14E,19Z-docosahexaenoic acid (Resolvin D2); MaR1: 7R,14S-dihydroxy-4Z,8E,10E,12Z,16Z,19Z-docosahexaenoic acid (Maresin-1); LXB4: (5S,6E,8Z,10E,12E,14R,15S)-5,14,15-Trihydroxyicosa-6,8,10,12-tetraenoic acid (LipoxinB4); LTB4: (5S,6Z,8E,10E,12R,14Z)-5,12-Dihydroxyicosa-6,8,10,14-tetraenoic acid (Leukotriene B4); 3HMYR: 3-Hydroxy-tetradecanoic acid; 16HPAL: 16-Hydroxy-hexadecanoic acid; 12HEST: 12-Hydroxyoctadecanoic acid. PGF2α: (Z)-7-[(1R,2R,3R,5S)-3,5-dihydroxy-2-[(E,3S)-3-hydroxyoct-1-enyl]cyclopentyl]hept-5-enoic acid (Prostaglandin 2α). IFC0 (stage 0 or control group without steatosis) IFC < 6.4%; IFC1 (stage 1 with mild steatosis) 6.4% ≤ IFC < 17.4%; IFC2 (stage 2 with moderate steatosis) 17.4% ≤ IFC < 22.1%; and IFC3 (stage 3 with severe steatosis) IFC ≥ 22.1.

**Table 3:** Correlation between the circulating levels of free fatty acids and oxylipins and the intrahepatic fat content or malondialdehyde levels.

		IFC (%)	MDA (nM)
AA (nM)	Cor.	**0.572**	**0.204**
	Sig.	0.000	0.023
EPA (nM)	Cor.	**0.424**	0.163
	Sig.	0.002	0.109
ETA (nM)	Cor.	**0.513**	0.103
	Sig.	0.003	0.298
15HETE (nM)	Cor.	**0.442**	**0.345**
	Sig.	0.001	0.001
17DoHE (nM)	Cor.	**0.488**	0.140
	Sig.	0.000	0.202
RvD2 (nM)	Cor.	0.019	**0.241**
	Sig.	0.897	0.024
MaR1 (nM)	Cor.	**0.307**	**0.446**
	Sig.	0.032	<0.001
LXB4 (nM)	Cor.	**0.289**	0.197
	Sig.	0.044	0.071
LTB4 (nM)	Cor.	0.231	**0.239**
	Sig.	0.110	0.028
3HMYR (nM)	Cor.	**0.286**	**0.260**
	Sig.	0.046	0.005
16HPAL (nM)	Cor.	**0.239**	**0.262**
	Sig.	0.050	0.013
12HEST (nM)	Cor.	**0.438**	**0.307**
	Sig.	0.002	0.001
PGF2α (nM)	Cor.	**0.241**	**0.227**
	Sig.	0.047	0.037
MDA (nM)	Cor.	**0.222**	-
	Sig.	0.023	-

Statistical analysis: Bivariate correlation Pearson test. Cor. indicates Pearson correlation coefficient. Sig. indicates statistical significance. Bold number indicates statistically significant *p* < 0.05. AA: 5,8,11,14-Eicosatetraenoic acid (Arachidonic acid); EPA: 5,8,11,14,17-eicosapentenoic acid; ETA: 8,11,14,17-eicosatetraenoic acid; 15HETE: 15-Hydroxy-5,8,11,13-Eicosatetraenoic acid; 17DoHE: 17-Hydroxy-4,7,10,13,16,19-docosahexaenoic acid; RvD2: 7S,16R,17S-trihydroxy-4Z,8E,10Z,12E,14E,19Z-docosahexaenoic acid (Resolvin D2); MaR1: 7R,14S-dihydroxy-4Z,8E,10E,12Z,16Z,19Z-docosahexaenoic acid (Maresin-1); LXB4: (5S,6E,8Z,10E,12E,14R,15S)-5,14,15-Trihydroxyicosa-6,8,10,12-tetraenoic acid (LipoxinB4); LTB4: (5S,6Z,8E,10E,12R,14Z)-5,12-Dihydroxyicosa-6,8,10,14-tetraenoic acid (Leukotriene B4); 3HMYR: 3-Hydroxy-tetradecanoic acid; 16HPAL: 16-Hydroxy-hexadecanoic acid; 12HEST: 12-Hydroxyoctadecanoic acid. PGF2α: (Z)-7-[(1R,2R,3R,5S)-3,5-dihydroxy-2-[(E,3S)-3-hydroxyoct-1-enyl]cyclopentyl] hept-5-enoic acid (Prostaglandin 2α); IFC: intrahepatic fat content; MDA: malondialdehyde.

**Table 4:** Correlation between the circulating levels of free fatty acids and oxylipins.

		AA (nM)	EPA (nM)	ETA (nM)	15HETE (nM)	17HDoHE (nM)	RvD2 (nM)	MaR1 (nM)	LXB4 (nM)	LTB4 (nM)	3HMYR (nM)	16HPAL (nM)	12HEST (nM)
EPA (nM)	Cor	0.962 **	--										
	Sig	<0.001											
ETA (nM)	Cor	0.963 **	0.930 **	--									
	Sig	<0.001	<0.001										
15HETE (nM)	Cor	0.248 *	0.159	0.118	--								
	Sig	0.015	0.124	0.254									
17HDoHE (nM)	Cor	0.208 *	0.164	0.124	0.805 **	--							
	Sig	0.043	0.113	0.232	<0.001								
RvD2 (nM)	Cor	0.121	0.093	0.051	0.191	0.137	--						
	Sig	0.247	0.275	0.625	0.065	0.187							
MaR1 (nM)	Cor	0.236 *	0.235 **	0.098	0.485 **	0.579 **	0.367 **	--					
	Sig	0.021	0.022	0.346	<0.001	<0.001	<0.001						
LXB4 (nM)	Cor	0.161	0.148	0.018	0.365 **	0.350 **	0.387 **	0.903 **	--				
	Sig	0.119	0.153	0.864	<0.001	0.001	0.001	<0.001					
LTB4 (nM)	Cor	0.161	0.193	0.013	0.090	0.507 **	0.107	0.347 **	0.310 **	--			
	Sig	0.119	0.061	0.904	0.319	<0.001	0.304	0.001	0.002				
3HMYR (nM)	Cor	0.205 *	**0.195**	0.033	0.364 **	0.335 **	0.445 **	0.870 **	0.949 **	0.337 **	--		
	Sig	0.046	0.058	0.750	<0.001	0.001	<0.001	<0.001	<0.001	0.001			
16HPAL (nM)	Cor	0.694 **	0.707 **	0.590 **	0.226 *	0.230 *	0.425 **	0.730 **	0.708 **	0.226 **	0.793 **	--	
	Sig	<0.001	<0.001	<0.001	0.028	0.025	<0.001	<0.001	<0.001	0.028	<0.001		
12HEST (nM)	Cor	0.396 **	0.366 **	0.194	0.645 **	0.551 **	0.320 **	0.786 **	0.823 **	0.501 **	0.835 **	0.682 **	--
	Sig	<0.001	<0.001	0.060	<0.001	<0.001	0.002	<0.001	<0.001	<0.001	<0.001	<0.001	
PGF2α (nM)	Cor	0.193	0.195	0.040	0.386 **	0.317 **	0.416 **	0.873 **	0.953 **	0.328 **	0.972 **	0.790 **	0.793 **
	Sig	0.061	0.058	0.703	<0.001	0.002	<0.001	<0.001	<0.001	0.001	<0.001	<0.001	<0.001

Statistical analysis: Bivariate correlation Pearson test. Cor. indicates Pearson correlation coefficient. Sig. indicates statistical significance. Bold number indicates statistically significant ** *p* < 0.01 and * *p* < 0.05. AA: 5,8,11,14-Eicosatetraenoic acid (Arachidonic acid); EPA: 5,8,11,14,17-eicosapentenoic acid; ETA: 8,11,14,17-eicosatetraenoic acid; 15HETE: 15-Hydroxy-5,8,11,13-Eicosatetraenoic acid; 17DoHE: 17-Hydroxy-4,7,10,13,16,19-docosahexaenoic acid; RvD2: 7S,16R,17S-trihydroxy-4Z,8E,10Z,12E,14E,19Z-docosahexaenoic acid (Resolvin D2); MaR1: 7R,14S-dihydroxy-4Z,8E,10E,12Z,16Z,19Z-docosahexaenoic acid (Maresin-1); LXB4: (5S,6E,8Z,10E,12E,14R,15S)-5,14,15-Trihydroxyicosa-6,8,10,12-tetraenoic acid (LipoxinB4); LTB4: (5S,6Z,8E,10E,12R,14Z)-5,12-Dihydroxyicosa-6,8,10,14-tetraenoic acid (Leukotriene B4); 3HMYR: 3-Hydroxy-tetradecanoic acid; 16HPAL: 16-Hydroxy-hexadecanoic acid; 12HEST: 12-Hydroxyoctadecanoic acid. PGF2α: (Z)-7-[(1R,2R,3R,5S)-3,5-dihydroxy-2-[(E,3S)-3-hydroxyoct-1-enyl]cyclopentyl]hept-5-enoic acid (Prostaglandin 2α).

**Table 5:** Diagnostic accuracy of plasma oxylipin or free fatty acid levels for grading hepatic steatosis.

	Steatosis Grade	AUCROC	*p*	95% Interval Confidence	Threshold (nM)	TPF (%)	FPF (%)
12HEST (nM)	IFC0 vs. ≥IFC1	0.572	0.338	0.425–0.718			
	≤IFC1 vs. ≥IFC2	0.661	0.014	0.535–0.788	12.3	83	72
	≤IFC2 vs. IFC3	0.694	0.047	0.477–0.910	30	80	53
	IFC0 vs. IFC3	0.690	0.110	0.439–0.942			
PGF2α (nM)	IFC0 vs. ≥IFC1	0.572	0.338	0.422–0.722			
	≤IFC1 vs. ≥IFC2	0.581	0.215	0.441–0.721			
	≤IFC2 vs. IFC3	0.748	0.011	0.537–0.958	0.675	80	14
	IFC0 vs. IFC3	0.731	0.050	0.494–0.968	0.665	78	16
15HETE (nM)	IFC0 vs. ≥IFC1	0.579	0.292	0.430–0.728			
	≤IFC1 vs. ≥IFC2	0.552	0.430	0.415–0.688			
	≤IFC2 vs. IFC3	0.689	0.050	0.484–0.895	0.675	80	14
	IFC0 vs. IFC3	0.696	0.099	0.464–0.927			
ETA (nM)	IFC0 vs. ≥IFC1	0.647	0.050	0.525–0.768	0.42	82	79
	≤IFC1 vs. ≥IFC2	0.573	0.267	0.434–0.711			
	≤IFC2 vs. IFC3	0.626	0.197	0.397–0.854			
	IFC0 vs. IFC3	0.661	0.176	0.390–0.932			

The discriminatory capability of plasma oxylipin or fatty acid concentrations for different steatosis grades was tested by using the following dichotomizations: IFC0 vs. IFC1 or greater (IFC0 vs. ≥IFC1); IFC1 or less vs. IFC2 or greater (≤IFC1 vs. ≥IFC2); IFC2 or less vs. IFC3 (≤IFC2 vs. IFC3); IFC0 vs. IFC3 (IFC0 vs. IFC3). For each set of dichotomized steatosis grades, the area under the receiver operating characteristic curve (AUCROC) was calculated. The oxylipins or fatty acids with an AUCROC significantly different from 0.5 were selected as parameters with discriminant capability. The lowest plasma oxylipin or fatty acid threshold value that provided 80% or greater specificity to distinguish between dichotomized steatosis grades was selected. At that oxylipin or fatty acid threshold value, the positive predictive value and negative predictive value to distinguish between dichotomized steatosis grades were calculated. AUCROC: area under the curve receiver operating characteristic; FPF: false positive fraction; IFC: intrahepatic fat content; TPF: true positive fraction; *p*: significance vs. 0.5 as non-discriminating AUCROC value. ETA: 8,11,14,17-eicosatetraenoic acid; 15HETE: 15-Hydroxy-5,8,11,13-Eicosatetraenoic; 12HEST: 12-Hydroxyoctadecanoic acid. PGF2α: (Z)-7-[(1R,2R,3R,5S)-3,5-dihydroxy-2-[(E,3S)-3-hydroxyoct-1-enyl]cyclopentyl]hept-5-enoic acid (Prostaglandin 2α). IFC0 (stage 0 or control group without steatosis) IFC < 6.4%; IFC1 (stage 1 with mild steatosis) 6.4% ≤ IFC < 17.4%; IFC2 (stage 2 with moderate steatosis) 17.4% ≤ IFC < 22.1%; and IFC3 (stage 3 with severe steatosis) IFC ≥ 22.1%.

## Data Availability

The data presented in this study are available on request from the corresponding author.
